# The Impact of Jump Type on Muscle Contractile Behavior: Fatigue or Potentiation After Countermovement and Stiffness Jumps?

**DOI:** 10.3390/sports13120437

**Published:** 2025-12-04

**Authors:** Vedran Dukarić, Ivan Bon, Marijo Baković

**Affiliations:** Department of Kinesiology of Sports, Faculty of Kinesiology, University of Zagreb, 10000 Zagreb, Croatia; ivan.bon@kif.unizg.hr (I.B.); marijo.bakovic@kif.unizg.hr (M.B.)

**Keywords:** tensiomyography, muscle contractile properties, post-activation potentiation, countermovement jump, stiffness jump, neuromuscular function

## Abstract

Jumping exercises are widely applied in sport performance and conditioning due to their crucial role in enhancing neuromuscular function and lower-limb power. Acute effects related to contractile properties measured by tensiomyography (TMG) remain insufficiently explored. This study aimed to examine the acute effects of two jump types—bilateral countermovement jumps (CMJs) and stiffness jumps (STs)—on the contractile properties of the vastus medialis (VM) and medial gastrocnemius (GM) muscles. Twenty-nine kinesiology students (fourteen males, fifteen females; age 19.4 ± 0.7 years) performed CMJ and ST protocols in a randomized order. Muscle contractile characteristics were measured before and immediately after each protocol and analyzed using a mixed-model repeated-measures ANOVA. Significant pre–post changes were found in both muscles. In the VM, contraction (Tc) and delay (Td) times decreased (*p* < 0.01), indicating faster responses, whereas relaxation time (Tr) increased and sustain time (Ts) decreased (*p* < 0.05), suggesting temporary fatigue. Maximal displacement (Dm) increased (*p* < 0.01), indicating reduced stiffness. In contrast, the GM showed greater responsiveness after stiffness jumps, characterized by shorter Tc and Td (*p* < 0.01), and reduced endurance after CMJs. These findings highlight muscle specific neuromuscular adaptations and provide practical insights for optimizing warm-up, training, and rehabilitation protocols through targeted jump selection.

## 1. Introduction

Muscle activity and responses to stimuli have been extensively studied in exercise physiology and sport science [1,2]. Various external and internal stimuli can lead either to muscle fatigue or to enhanced activation of muscle fibers. Understanding the acute mechanical responses of skeletal muscle following high-intensity activities such as jumps, heavy resistance exercises, or plyometric drills is essential for interpreting mechanisms like post-activation potentiation (PAP) and for designing warm-up or conditioning strategies that enhance explosive performance [3].

Jumping tasks are among the most widely used methods for developing lower-limb strength and neuromuscular potentiation. Commonly employed jump types—including the squat jump (SJ), countermovement jump (CMJ), stiffness jump (ST), and drop jump (DJ)—reflect distinct patterns of muscle activation and neural recruitment. The CMJ incorporates the stretch-shortening cycle (SSC), which improves contraction efficiency through elastic energy storage and reflexive activation [4]. Similarly, the stiffness jump, performed mainly from the feet, also involves the SSC but emphasizes the ankle joint’s contribution, maintaining knee stiffness so that movement is executed predominantly through the foot–ankle complex. The CMJ involves eccentric–concentric action dominated by knee extensors (VM), while the ST minimizes knee involvement and emphasizes reactive stiffness in the ankle and foot, which engage the GM. Therefore, comparisons between these two jump strategies can provide a model to test how muscles with different roles in the kinetic chain respond differently to acute plyometric loading. Because these jumps engage different muscular and neural mechanisms, they likely induce specific acute adaptations in contractile behavior and fatigue resistance. Their frequent inclusion in pre-activation routines before explosive actions such as sprinting and jumping further highlights their applied importance. The SSC, which underlies most plyometric movements, enhances concentric force output through elastic energy storage and reflex potentiation, making jump-based tasks particularly suitable for studying acute neuromuscular responses [5,6].

To investigate these phenomena, non-invasive methods for muscle assessment have become increasingly valuable. One such technique is tensiomyography (TMG), which quantifies mechanical muscle responses to controlled electrical stimulation [7,8]. Key parameters assessed by TMG include the contraction time (Tc), relaxation time (Tr), maximal displacement (Dm), and contraction velocity [8,9]. These measures provide insight into the muscle fiber composition, functional state, and potential imbalances both within and between muscles [10,11]. As an electrostimulation-based tool, TMG enables objective evaluation of neuromuscular performance, whereas dynamic tasks such as jump execution remain essential for understanding activation patterns under functional load [12]. Previous research [13] presented statistically significant correlations between power assessment variables of the CMJ and radial displacement with the use of TMG (r = −0.80; *p* < 0.01).

Systematic reviews and methodological analyses indicate that Tc and Dm are among the most reliable TMG-derived variables, offering valuable information for monitoring muscle adaptations and detecting intermuscular differences [7,14]. Therefore, the present study aimed to characterize acute pre-to-post changes in TMG parameters (Td, Tc, Ts, Tr, and Dm) of the vastus medialis and medial gastrocnemius following sets of vertical jumps performed with and without eccentric knee action. This approach provides an insight to isolate muscle-specific mechanical adaptations and to determine whether different jump modalities preferentially activate proximal (knee-dominant) or distal (ankle-dominant) segments of the kinetic chain. It has been proven that prolonged exercise can induce a significant loss in performance [15] after CMJ of the VM muscle. Thus, it needs to be investigated whether the CMJ and ST in different conditions induce greater activation or fatigue. Furthermore, eccentric plyometric programs are proven to increase strength, speed, and TMG parameters, rather than exercises focused on concentric contraction [16], as eccentric actions induce significant performance enhancement, and different conditioning (jump types) may differentially influence contractile properties [17]. Thus, defining exercises that can induce positive effects for certain muscles, such as PAP, need to be investigated. In addition, understanding these short-term neuromechanical adjustments could help refine warm-up and potentiation strategies in sports requiring explosive strength, agility, and stretch-shortening efficiency.

Based on the mentioned it is hypothesized that both jump types would acutely modify muscle contractile properties, with the CMJ producing greater effects in the vastus medialis due to increased knee involvement, and the ST inducing faster responses in the gastrocnemius due to predominant ankle activation.

## 2. Materials and Methods

### 2.1. Sample of Participants

This research employed a repeated-measures experimental design in which all participants completed both jump conditions (CMJ and ST) in randomized order, with pre and post measurements taken for each muscle. The sample consisted of first-year kinesiology students from the Faculty of Kinesiology, University of Zagreb. A total of 29 participants were included in this study (mean age 20.08 ± 1.12 yr; height 174.70 ± 10.70 cm; body mass 71.34 ± 13.70 kg), comprising 14 males (19.83 ± 0.79 yr; 182.80 ± 5.16 cm; 80.41 ± 11.07 kg) and 15 females (20.34 ± 1.38 yr; 166.10 ± 7.87 cm; 61.62 ± 8.70 kg). Based on a G*Power (v3.1.9.7) analysis, the required sample size was calculated as N = 28, assuming an effect size of 0.25, a significance level of 0.05, a statistical power of 0.80, two conditions (CMJ and ST), and four dependent variables. The inclusion criteria implied that participants were free of neuromuscular or orthopedic impairments for at least six months and engaged in regular sport activity at least three times per week. Menstrual cycle phase was not controlled, which we acknowledge as a methodological limitation given potential cycle-related fluctuations in neuromuscular function. Also, knowledge of and familiarity with the observed jump were requirements for inclusion in the research. The exclusion criteria were the existence of an acute injury, any contraindication to neuromuscular electrical stimulation, and inability to complete the jump protocols as instructed. The study was conducted in accordance with the Declaration of Helsinki and approved by the Ethics Committee of the Faculty of Kinesiology, University of Zagreb (Approval No. 85/2024).

### 2.2. Instruments

Body height was measured using a SECA stadiometer (SECA, Hamburg, Germany), and body composition was assessed using a TANITA BC-545n bioelectrical impedance analyzer (Tanita, Tokyo, Japan). Muscle contractile properties were assessed with tensiomyography (TMG; TMG-BMS Ltd., Ljubljana, Slovenia). The device consists of an electrical stimulator and a digital displacement transducer that measures changes in muscle belly thickness during contraction. The TMG system allows high-precision detection of latency, contraction, and the relaxation velocity, as well as the maximal contraction amplitude. During testing, the displacement sensor was positioned perpendicularly to the skin over the thickest point of the muscle belly, while two self-adhesive electrodes were placed proximally and distally to the sensor tip. Several validation studies have confirmed the accuracy and reliability of TMG measurements [9,11,18,19].

### 2.3. Sample of Variables

**Figure 1:**
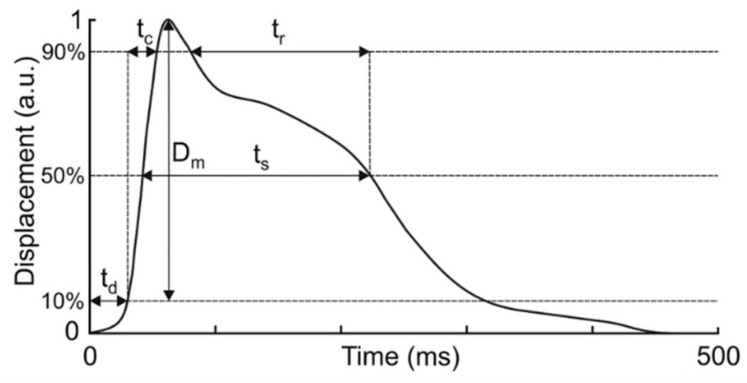
Time and displacement curve with observed variables. Retrieved from https://www.tmg-bodyevolution.com/research/tmg-research-measuring-device/ (accessed on 6 October 2025).

Five primary TMG variables were analyzed: the delay time (Td), contraction time (Tc), sustain time (Ts), relaxation time (Tr), and maximal displacement (Dm). Td represents the time between the electrical impulse and 10% of the contraction; Tc is the time between 10% and 90% of maximal contraction; Ts is the time between 50% of contraction and 50% of relaxation; Tr represents the time between 90% and 50% of relaxation; and Dm reflects the maximal amplitude of muscle displacement (Figure 1). Among these parameters, Dm and Tc are generally considered the most valid indicators of muscle contractile behavior [18,19]. Dm provides information about muscle stiffness, fatigue, and early atrophic processes [20,21,22], while Tc is often associated with the muscle fiber type composition and rate of force development [9,18,23]. Td, representing the reaction or activation time, has been used as an indicator of neuromuscular responsiveness [24].

Jump performance was analyzed with the use of an Optojump (Microgate, Bolzano, Italy) measurement device. The variables observed from this device were CMJ_tcont—duration of contact time during performance of repetitive CMJs; CMJ_H—countermovement jump height; CMJ_RSI (mod)—modified reactive strength index; ST_tcont—duration of contact time during performance of repetitive stiffness jumps; ST_H—stiffness jump height; and ST_RSI (mod)—stiffness jump modified reactive strength index.

### 2.4. Testing Procedure

**Figure 2:**
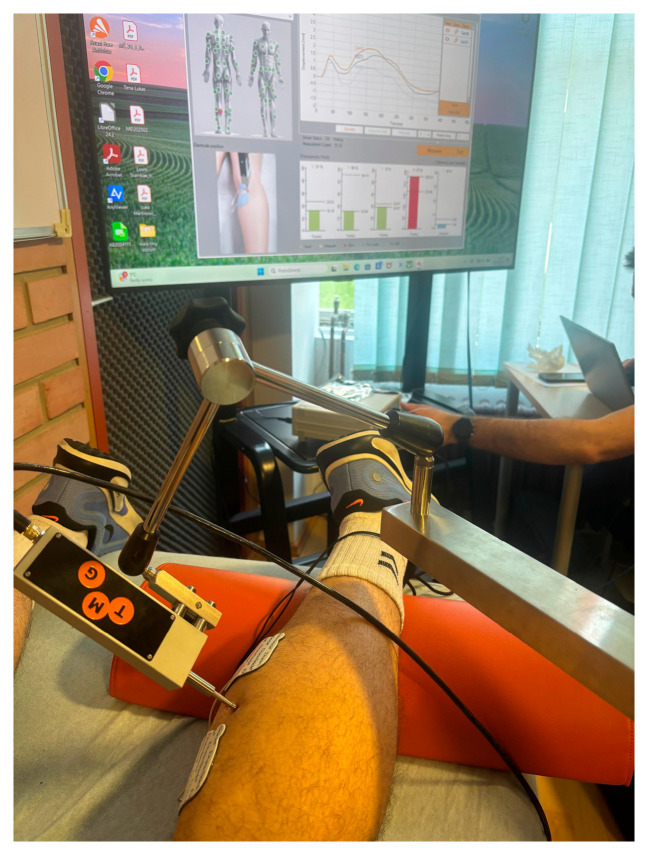
Sensor positioning and measurement of GM muscle.

Prior to testing, participants were familiarized with the protocol and potential risks. They performed a standardized warm-up consisting of basic movement patterns, dynamic stretching, and specific preparatory drills replicating the tested movements. Following the warm-up, TMG measurements were taken from the vastus medialis and medial gastrocnemius muscles in a relaxed supine position. The muscle belly was palpated during voluntary contraction to identify the thickest point for sensor placement. The positive electrode (anode) was placed proximally and the negative electrode (cathode) distally, with both electrodes self-adhesive (Figure 2). The inter-electrode distance was standardized at 5 cm, positioned proximally and distally to the probe. Electrical stimulation began at 20 mA and increased in 10 mA increments until the maximal radial displacement plateaued (typically 60–90 mA). A single-pulse square wave (1 ms duration) was used. The displacement sensor was calibrated before each session according to the manufacturer’s instructions.

After the baseline measurement, participants performed 10 bilateral countermovement jumps (CMJs) with controlled amortization in the vertical plane, which was monitored visually and controlled by ensuring no excessive knee valgus or trunk lean. Immediately following the jumps, post-test TMG measurements were obtained using identical procedures. A standardized passive recovery period of 5 min was implemented between the CMJ and ST protocols. After completion of the CMJ protocol and full recovery, participants performed bilateral stiffness jumps (STs), executed on the spot with minimal knee flexion to emphasize the ankle–foot contribution. Participants were instructed to maintain knee flexion at <15°, and trials were visually controlled to ensure correct performance. Any jump displaying excessive knee bend was repeated. During the jump protocols, arm swing was permitted to maximize the potential of the jump. The same measurement procedures were used for both muscles and both jump conditions. On each muscle, 3–5 single stimuli were delivered, and the largest reproducible response (≤5% variation) was retained for analysis.

### 2.5. Data Analysis

All data analyses were conducted using the Statistica software package (v14.01.25; TIBCO Software Inc., San Ramon, CA, USA). Data normality was verified using the Shapiro–Wilk test. Basic descriptive statistics were calculated for all variables. Additionally, descriptive parameters of jump performance were calculated and are reported. Differences between pre and post measurements were determined using repeated-measures ANOVA, with the level of significance set at *p* < 0.05. The effect size for the ANOVA is reported using partial eta squared (η^2^), which represents the proportion of total variance in the dependent variable that can be attributed to a given factor, providing an estimate of the practical significance of observed effects.

## 3. Results

This study examined pre- to post-intervention changes in the contractile properties of the vastus medialis (VM) and medial gastrocnemius (GM) muscles after two jump protocols: countermovement jump (CMJ pre vs. post) and stiffness jump (ST pre vs. post). Table 1 presents the descriptive statistics of jump performance variables obtained during the countermovement jump (CMJ) and stiffness jump (ST) protocols. For the CMJ, participants demonstrated an average contact time of 0.49 ± 0.10 s, a mean jump height of 32.20 ± 7.47 cm, and an RSI of 0.70 ± 0.28. In contrast, during the ST, the contact time was much shorter (0.18 ± 0.03 s), while the average jump height was 24.63 ± 8.73 cm, and the RSI was higher at 1.37 ± 0.34, indicating greater reactive strength efficiency due to shorter ground contact phases. The minimum and maximum values in each variable highlight the variability among the participants, reflecting differences in power, elasticity, and reactive ability between individuals and between jump types.

The first analysis (CMJ condition pre vs. post) refers to changes in the VM after CMJ performance, while the second (ST condition pre vs. post) describes responses after ST performance (Table 2).

The analysis revealed significant pre-to-post changes in all VM variables after the CMJ protocol. Specifically, Tc, Ts, and Td decreased, indicating faster contraction and reduced contraction duration, while Tr and Dm increased, suggesting slower relaxation and lower muscle stiffness. Following the ST protocol, a similar distribution of changes was observed, with significant differences in four out of five variables (all except Ts). Thus, both jump types induced measurable alterations in VM contractile behavior, with the CMJ producing slightly broader changes across parameters. The GM exhibited a different pattern of response compared with the VM (Table 3).

## 4. Discussion

The main purpose of this study was to examine the acute effects of different jump types (CMJ and ST) on contractile properties of the vastus medialis (VM) and medial gastrocnemius (GM) using tensiomyography (TMG). It has previously been proven that plyometric-based training reliably enhances SSC efficiency and jump performance across various athletic populations [25]. Similarly, the differential activation demands between the CMJ and ST observed here emphasize that the proximal (knee-dominant) and distal (ankle-dominant) musculature respond differently depending on task mechanics [26]. The jump performance is clearly presented in Table 1. The CMJ produced a longer contact time (0.49 ± 0.10 s), higher jump height (32.20 ± 7.47 cm), and lower RSI (0.70 ± 0.28), which is characteristic of a slow stretch-shortening cycle (SSC) task dominated by knee extensor involvement. These values correspond well to normative CMJ performance in recreational adults and are supported by the literature indicating that slow SSC actions rely heavily on eccentric–concentric coordination and knee-dominant propulsion [4]. In contrast, the stiffness jump exhibited very short contact times (0.18 ± 0.03 s), moderate jump heights (24.63 ± 8.73 cm), and a higher RSI (1.37 ± 0.34), all of which indicate a fast SSC strategy relying primarily on ankle stiffness [5]. The results demonstrated that both the VM and GM exhibited significant alterations after the jump protocols, but the pattern of change differed between muscles and conditions. Specifically, the VM showed reduced contraction and delay times alongside increased relaxation time and maximal displacement, while GM adaptations varied, decreasing the contraction time after STs and the sustain time after CMJs. Furthermore, in the GM, increased values of Dm after CMJs and decreased delay times after STs were observed. A similar trend for the VM muscle [27] was determined, with the biggest differences obtained in the Tr parameter.

### 4.1. Vastus Medialis (VM)

As previously mentioned, the VM muscle reacted significantly to the jump stimulus except in the Ts variable after the second effect (ST). Tc decreased significantly across both effects (first effect: 24.00 to 22.14 ms, *p* < 0.01; second effect: 24.49 to 21.72 ms, *p* < 0.01). This reduction indicates a shift towards faster contractile behavior. This finding suggests that repetitive jumps in both conditions led to increased recruitment or potentiation of fast-twitch fibers. Ts presented a decrease in the CMJ (pre vs. post) and an equal result after the ST (pre vs. post). Since there was an increase in Dm, it can be concluded that the Ts variable remained or was quicker after jumps, which demonstrates a positive reaction of the muscle to the stimulus. Relaxation time (Tr) exhibited a significant increase in both effects (CMJ pre vs. post: 80.11 to 103.22 ms, *p* = 0.03; effects pre vs. post: 72.22 to 115.58 ms, *p* < 0.01). Prolonged Tr is associated with the level of fatigue, which affects the relaxation time. The presented results displayed higher Tr values after the ST (pre vs. post). This can be explained from a technical perspective of jump performance. Even though in CMJs there is a greater load on the VM muscle, during the performance of repetitive jumps from the feet, a high demand was set on keeping the knees straight, which resulted in greater isometric muscle activation and more relaxation time. Dm increased significantly in both effects, indicating greater post-jump activation and contraction. The presented results showed more of an effect after STs (7.77 to 9.24 mm). Td also decreased significantly, reflecting improved neuromuscular responsiveness and more efficient coupling. Shorter Td values imply a faster onset of muscle contraction after stimulation, highlighting acute potentiation of the neuromuscular system. The presented research examined a recreational student sample; the post-jump TMG values for the VM (Tc and Dm) can be compared with established normative values obtained in elite-level players. The VM in professional soccer players shows a mean Tc of 25.3 ms and Dm of 7.45 mm. The relative differences between our acute post-jump responses and these normative benchmarks offer insight into the magnitude and potential functional relevance of jump-induced contractile modulation, particularly considering training status and muscle conditioning [28].

### 4.2. Gastrocnemius Medialis (GM)

The gastrocnemius medialis showed a lower sensitivity to both the CMJ and ST interventions compared with the vastus medialis. The first effect (CMJ pre vs. post) induced significant changes in displacement- and sustain-related parameters, while the second effect (ST pre vs. post) primarily influenced contraction and delay times. Contraction time (Tc) showed no significant change in the CMJ pre vs. post (21.26 to 20.92 ms, *p* = 0.61) but decreased significantly in the ST pre vs. post (23.05 to 20.68 ms, *p* < 0.01). This reduction reflects faster muscle contractility and suggests acute potentiation of fast-twitch fibers and improved contractile speed after repeated ST performance. With a similar distribution to that of the VM, Ts of the GM decreased significantly in the first effect (172.61 to 140.82 ms, *p* = 0.03), indicating a reduced ability to maintain contraction and reflecting acute fatigue or diminished endurance capacity in the GM after repeated CMJ execution. No significant difference was observed in the ST pre vs. post (175.92 to 156.14 ms, *p* = 0.18). The relaxation time (Tr) did not change significantly with either effect. Even though there was no statistical significance, Tr was reduced after jump performance. Dm increased significantly in the CMJ pre vs. post (2.38 to 2.61 mm, *p* = 0.03) but showed no difference in the ST pre vs. post (2.55 to 2.60 mm, *p* = 0.60). Increased Dm indicates reduced stiffness and greater contraction of the GM immediately after jump execution. This adaptation may enhance elastic energy storage in subsequent SSC actions, although it may also reflect reductions in passive muscle tone. Td remained unchanged in the CMJ but decreased significantly in the ST. Shorter Td indicates improved contraction efficiency and greater neuromuscular responsiveness, likely reflecting acute potentiation effects after higher-intensity loading.

The results presented display effects of two types of jumps commonly used in sports to improve acute performance. The VM results present significant improvement after the performance of both interventions. The GM results have a similar trend but a lot less potentiation. These findings align with the mechanisms of post-activation potentiation (PAP), whereby a conditioning stimulus (e.g., maximal jumps) enhances subsequent muscle contractility via phosphorylation of myosin regulatory light chains and increased recruitment of higher-threshold motor units [3,29]. Moreover, the pattern of potentiation and low-level fatigue presented in both muscles reflects the interaction between PAP and fatigue described by recent work examining session dose and training frequency in jump athletes, where higher-intensity SSC actions acutely enhance contractile speed but simultaneously result in neuromuscular strain [30]. Previous studies have shown similar reductions in Tc measured with TMG after short-term high-intensity interventions, supporting the idea that TMG is sensitive to contractile potentiation [17,20]. Alongside potentiation indicators, several results suggested the presence of acute fatigue. In the VM, the sustain time (Ts) decreased significantly, reflecting reduced ability to maintain contraction and potentially impaired fatigue resistance. The concurrent increase in relaxation time (Tr) further supports this interpretation, as prolonged relaxation is often observed when muscles are metabolically stressed or fatigued [31]. This dual presence of potentiation (faster Tc, Td) and fatigue (reduced Ts, increased Tr) aligns with theoretical models of interaction between PAP and fatigue in determining performance outcomes [32]. The balance between these two processes likely depends on the jump type, volume, and recovery intervals. Reduced stiffness may facilitate greater muscle lengthening and storage of elastic energy during subsequent SSC actions, potentially enhancing jump or sprint performance in the short term [4]. However, lower stiffness could also indicate transient decreases in passive tone and structural support, which may increase injury risk under certain conditions [21]. Overall, these findings suggest the use of STs as they tend to have slightly better results in terms of potentiation and do not prolong delay and relaxation times. This highlights the need for careful consideration of the jump modality and volume when jumps are used as part of potentiation or warm-up routines.

### 4.3. Limitations

While the present study demonstrates that TMG is sensitive to acute changes in muscle contractile properties after jumping, its limitations must be acknowledged. Several studies have highlighted that TMG may not always detect subtle individual changes in fatigue or performance, especially in elite populations, and results can be influenced by electrode placement, measurement reliability, and muscle depth [17,32]. Moreover, the sample consisted of recreationally active kinesiology students with heterogeneous training backgrounds, and the findings may not be directly generalizable to competitive or elite athletic populations whose neuromuscular profiles and fatigue–potentiation responses significantly differ. Regarding female participants, menstrual cycle phase was not recorded, which may have influenced TMG-derived contractile variables. Furthermore, as the main findings indicated an increase in performance, specifically potentiation and not fatigue, there is a need to discover how many sets or repetitions are required before the muscle becomes more fatigued and a drop in performance is detected.

## 5. Conclusions

This study aimed to determine the acute effects of two types of jumps—countermovement jumps (CMJs) and stiffness jumps (STs)—on the contractile properties of the vastus medialis (VM) and medial gastrocnemius (GM) muscles using tensiomyography (TMG). The results revealed that both jump types induced significant changes in muscle contractile behavior, but the magnitude and pattern of adaptation differed between the two muscles.

In the VM, both CMJs and STs led to shorter contraction (Tc) and delay (Td) times, indicating enhanced contractile speed and neuromuscular responsiveness. Increased maximal displacement (Dm) and prolonged relaxation time (Tr) suggested the presence of post-activation potentiation accompanied by mild fatigue. In contrast, the GM showed a more selective response: CMJs primarily affected the sustain time (Ts) and Dm, while STs produced greater reductions in Tc and Td, highlighting faster activation and improved efficiency of the gastrocnemius following ankle-dominant jumps.

Overall, both jump modalities elicited acute neuromuscular adaptations consistent with post-activation potentiation mechanisms. However, stiffness jumps produced greater potentiation effects without prolonging relaxation or delay times, suggesting that this jump type proves to be greater for inducing short-term improvements in contractile readiness. These findings emphasize the importance of selecting jump types according to specific training or warm-up objectives, as different movements elicit distinct muscular responses. This study provides preliminary insight into the acute contractile behavior of the VM and GM following two commonly used jump modalities. Although both CMJs and STs elicited measurable pre–post changes in TMG parameters, these effects were generally modest and likely reflect short-term neuromuscular adjustments rather than definitive evidence of potentiation or enhanced readiness. Differences between jump types appear to be muscle-specific rather than indicative of the superiority of one exercise over the other.

Future research should further explore the balance between potentiation and fatigue across multiple sets, varying intensities, and populations with different training backgrounds to optimize the practical application of TMG-based assessments and jump protocols in sports performance and rehabilitation. Thus, while this work contributes to the growing understanding of acute muscle responses assessed by TMG, the practical implications remain exploratory. Future studies using more ecologically valid tasks, sport-relevant performance measures, and highly trained populations are needed to clarify how different jump modalities influence contractile behavior and whether these acute responses translate into meaningful changes in performance.

## Figures and Tables

**Table 1:** Descriptive statistics of performance variables during countermovement (CMJ) and stiffness (ST) jump protocols.

Variable	Mean	Min	Max	St.Dev.
CMJ_tcont	0.49	0.29	0.79	0.10
CMJ_H	32.20	20.50	48.28	7.47
CMJ_RSI (mod)	0.70	0.34	1.56	0.28
ST_tcont	0.18	0.14	0.26	0.03
ST_H	24.63	13.10	52.98	8.73
ST_RSI (mod)	1.37	0.82	2.09	0.34

Legend: CMJ_tcont—contact time while performing repetitive CMJs; CMJ_H—height of jump; CMJ_RSI (mod)—modified reactive strength index; Stiff_tcont—contact time while performing repetitive stiffness jumps; Stiff_H—ST height; Stiff_RSI (mod)—modified ST reactive strength index; Mean—average value; Min—minimum value; Max—maximum value; St.Dev.—standard deviation.

**Table 2:** Differences in vastus medialis (VM) contractile parameters after CMJ (pre vs. post) and ST (pre vs. post).

CMJ (Pre vs. Post)-F = 6.75; *p* < 0.01; η^2^ = 0.58; df = 28						ST (Pre vs. Post)-F = 16.59; *p* < 0.01; η^2^ = 0.78; df = 28					
Var	Mean 1	Mean 2	−95.00%	+95.00%	*p*	Var	Mean 1	Mean 2	−95.00%	+95.00%	*p*
Tc	24.00	22.14	0.93	2.80	0.00 *	Tc	24.49	21.72	1.85	3.69	0.00 *
Ts	195.66	166.77	10.71	47.08	0.00 *	Ts	172.87	172.77	−14.21	14.40	0.99
Tr	80.11	103.22	−43.28	−2.95	0.03 *	Tr	72.22	115.58	−63.40	−23.33	0.00 *
Dm	8.07	9.15	−1.63	−0.55	0.00 *	Dm	7.77	9.24	−1.99	−0.96	0.00 *
Td	22.88	22.18	0.17	1.24	0.01 *	Td	22.85	21.60	0.93	1.59	0.00 *

Legend: Tc—contraction time; Ts—sustain time; Tr—relaxation time; Dm—maximal displacement; Td—delay time; Mean 1—average value of 1st measurement; Mean 2—average value of 2nd measurement; F—f statistic value; *p*—significance level; *—marked values were significant when *p* < 0.05.

**Table 3:** Differences in gastrocnemius medialis (GM) contractile parameters after CMJ (pre vs. post) and ST (pre vs. post).

CMJ (Pre vs. Post)-F = 6.07; *p* < 0.01; η^2^ = 0.56; df = 28						ST (Pre vs. Post)-F = 4.84; *p* < 0.01; η^2^ = 0.50; df = 28					
Var	Mean 1	Mean 2	−95.00%	+95.00%	*p*	Var	Mean 1	Mean 2	−95.00%	+95.00%	*p*
Tc	21.26	20.92	−1.00	1.67	0.61	Tc	23.05	20.68	0.84	3.91	0.00 *
Ts	172.61	140.82	3.48	60.09	0.03 *	Ts	175.92	156.14	−6.87	35.77	0.18
Tr	51.82	49.56	−20.52	25.02	0.84	Tr	62.22	47.91	−9.54	38.14	0.23
Dm	2.38	2.61	−0.46	−0.01	0.03 *	Dm	2.55	2.60	−0.25	0.15	0.60
Td	19.91	19.57	−0.34	1.00	0.32	Td	20.62	19.43	0.52	1.85	0.00 *

Legend: Tc—contraction time; Ts—sustain time; Tr—relaxation time; Dm—maximal displacement; Td—delay time; Mean 1—average value of 1st measurement; Mean 2—average value of 2nd measurement; F—f statistic value; *p*—significance level; *—marked values were significant when *p* < 0.05.

## Data Availability

The data presented in this study are available on request from the corresponding author due to privacy and ethical reasons.

## Abbreviations

The following abbreviations are used in this manuscript:

CMJ	Countermovement jump
ST	Stiffness jump
TMG	Tensiomyography
VM	Vastus medialis
GM	Gastrocnemius medialis
SSC	Stretch-shortening cycle
Td	Delay time
Tc	Contraction time
Ts	Sustain time
Tr	Relaxation time
Dm	Displacement
ms	Milliseconds
